# Arthroscopic mgmt of thumb CMCJ and STTJ arthritis

**DOI:** 10.1186/1753-6561-9-S3-A52

**Published:** 2015-05-19

**Authors:** Clara Wong Wing-Yee

**Affiliations:** 1 Department of Orthopaedics, Prince of Wales Hospital, Hong Kong

## 

Thumb CMCJ arthritis is a commonly encountered condition. A wide variety of surgical interventions are advocated to those who failed conservative measures. They include ligament reconstruction, osteotomy, resection arthroplasty, suspension arthroplasty, arthrodesis, prosthetic replacement, joint denervation and etc. They are tailored to different radiographic stages of disease, patient’s demand and expectations. However, it is well known that there is a poor correlation between severity of degenerative disease and clinical symptomatology. It is not uncommon to encounter patients having Eaton stage IV CMCJ1 arthritis with minimal symptoms.

What determines the magnitude of patient’s symptoms and functional disability with thumb CMCJ arthritis? Is it the degree of degenerative synovitis, severity of inflammation, mechanical instability, mechanical bone pain, or individual’s thumb biomechanics? And, what determines the surgical outcomes in each operation. In fact, the results of the above surgical interventions are variable and sometimes unpredictable. Is there a role of less invasive operation while giving a reliable outcome?

Arthroscopic synovectomy, debridement, with minimal bone work, of thumb CMCJ & STTJ arthritis, is a minimal invasive procedure, with both diagnostic and therapeutic role. It gives an excellent visualisation of the joint, including the joint capsule, cartilage, ligament, degree of synovitis, and etc. It allows the detection of any intra-articular changes long before they would be noted through routine radiographs, and enables earlier treatments in those patients. It provides better judgement of management decision according to the intra-articular pathology. Most important of all, it is a viable option to relieve patient’s symptoms.

From 2002 to 2013, 76 thumb CMCJ arthroscopic debridement and synovectomy were performed in 65 patients. There were 51 females. The average age was 56. The dominant hand was affected in 38 thumbs. All failed conservative measures for more than 6 months. The follow-up duration was 59 months on average. There were 38 thumbs of Eaton & Littler Stage II disease, 36 Stage III, one stage I and one stage IV. Most of them were operated under portal site local anaesthesia except two were under intravenous local anaesthesia and one was under forearm intravenous regional anaesthesia.

The average duration of operation was 68 minutes. 49 of them were classified as Badia’s Stage III arthroscopic changes, 23 Stage II, and 4 Stage I. Synovectomy and debridement of frayed joint capsule and radio-frequency ablation were done in all thumbs. Loose bodies were removed in 25 thumbs. Thermal shrinkage was done in 30 thumbs with intact, non-frayed ligaments. Limited bone work was done. Bone surface was smoothened and prominent osteophytes were removed in 12 thumbs. For those who received thermal shrinkage, they were immobilised in a cast for 4 weeks.

In an average follow up of 59 months, 69 cases experienced pain relief after the operation, occurring on average of 3 months after the surgery. The visual analogue pain score decreased from a mean 8.3/10 to 2.7/10. 20 patients had excellent final outcome in which pain relieved for >90% and lasted till the end of the follow-up. 14 had good results in which pain was relieved for >70% and lasted till the end of the follow-up. 24 had fair results in which pain was relieved for >70% and lasted for >1year or pain was relieved <=70% but was not a nuisance.

The grip strength was 88.3% of the contralateral side, whereas pinch power was 81.7%. The average Kapandji index was 8. DASH score averaged at 26.5. No complications was encountered. 7 patients requested the same operation on the contralateral thumb. 3 patients requested repeating the operation on the same thumb as pain relief lasted for 1-3 years. The overall patient satisfaction rate was 77%.

However, 7 cases never experienced pain relief after the operation. 18 patients had pain reappearing to the pre-operative level subsequently undergoing open surgeries at an average of 14 months (5 months to 4 years) after the arthroscopy.

Statistical analysis revealed that patient’s age, gender, duration of symptoms, MCPJ hyper-extendibility, radiological staging, size of the impinging osteophytes, cartilage status, loose body removal and post-operative radiological progression did not correlate with the outcome after arthroscopic debridement and synovectomy. Better outcomes were seen in those who had smoothening of the bone surface and prominent osteophytes removal, thermal shrinkage procedure, and post-operative immobilisation.

Removal of synovitis and frayed capsule might eliminate one of the source of pain. Debridement and the use of radio-frequency could be a kind of joint denervation to abolish pain transmission. Thermal shrinkage, postoperative immobilisation and postoperative joint fibrosis promoted stabilisation of the CMCJ. Limited bone work might help to promote bone bleeding and release of mesenchymal cells to the joint. We did arthroscopic partial trapeziectomy under portal site local anaesthesia on other occasions. Those patients did not anticipate pain during bone burring. Bare bone surface may not be the source of pain. We advocate limited bone work, without hemi-trapeziectomy, in order to preserve the hard subchondral bone, which may be helpful in sharing the load across the CMCJ.

Arthroscopic synovectomy, debridement, with minimal bone work in thumb CMCJ and STTJ arthritis, is a “buy time surgery” helping patients to endure the painful period of osteoarthritis, until the joint is partially stabilised naturally, the joint biomechanics are adjusted and adapted to the daily activities. It may also be a definitive procedure to alleviate symptoms. We regard this as the first line of surgical treatment in all stages of arthritis at the thumb CMCJ and STTJ.

